# Boutonniere Deformity of the Proximal Interphalangeal Joint in a Young, Active Female Patient: A Nonstandard Surgical Procedure

**DOI:** 10.7759/cureus.100032

**Published:** 2025-12-24

**Authors:** Jakub Florek, Oles Petrovych, Filip Georgiew, Patryk Kawa, Sebastian Janowiec

**Affiliations:** 1 Department of Orthopaedics and Traumatology, Rydygier Hospital, Brzesko, POL; 2 Faculty of Health Science, University of Applied Science, Tarnów, POL

**Keywords:** boutonniere, deformity, degenerative disease, flexion contracture, hand, pip, proximal interphalangeal joint, surgical treatment

## Abstract

One of the key elements of post-traumatic osteoarthritis treatment is not only pain relief but also the return of joint function close to pre-injury levels. This is particularly important for young and physically active individuals. Unfortunately, the ability to return to satisfactory proximal intraphalangeal joint mobility after surgical treatment is often limited. This article describes the treatment of a young, active female patient who, several years ago, suffered a proximal interphalangeal (PIP) joint injury in her fifth finger following a fall from a height. Initially, the patient was treated at another hospital using open reduction internal fixation and stabilization with two Kirschner wires. A complication of this treatment was a "boutonniere" finger deformity with stiffening of the phalanx in the position of flexion contracture of 80° and the presence of finger pain with a numerical rating scale (NRS) score of 6. X-ray examination revealed initial degenerative changes in the joint surface of the proximal phalanx. The patient was classified for joint replacement using a cementless CapFlex hemiarthroplasty. A follow-up examination was performed after six months, which revealed an unsatisfactory clinical outcome because the total range of motion was 10° (extension deficit: 80°; flexion range: 90°) and the boutonniere deformity was still present. Pain intensity was 0 on the NRS scale. Due to the patient's active lifestyle, a joint decision was made to qualify the patient for revision surgery using a 3S ORTHO semiconstrained prosthesis. A follow-up examination performed eight weeks after the surgery showed correction of the butenoid deformity, improvement of the flexion range of motion to 50°, with limitation of the active extension movement (extension deficit reached 15°). Pain intensity was rated 0 on the NRS scale. After the follow-up examination, the patient was referred for additional specialized physiotherapy treatments, including post-isometric muscle relaxation, manipulation exercises, and whirlpool massage, to further improve the range of motion and dexterity of the treated hand. Additional physiotherapy treatments resulted in further improvement in range of motion and functional efficiency. Arthroplasty of the PIP joint using a cementless CapFlex hemiarthroplasty corrected the boutonniere deformity. At follow-up, a significant degree of flexion contracture developed. Revision surgery involving implantation of a 3S ORTHO prosthesis improved the flexion range of motion. The extension range of motion did not return to normal. According to the patient, the regained hand function met her expectations and allowed her to return to her previously performed activities. These results reflect the early follow-up period.

## Introduction

Boutonniere deformity describes a pathology in which the finger is flexed at the proximal interphalangeal (PIP) joint, and there is hyperextension at the distal interphalangeal (DIP) joint. This is usually a result of trauma and is caused by force applied to the top of a bent middle joint of a finger. A boutonniere deformity results when the triangular ligament and the central slip of the extensor tendon of a digit are disrupted [1].

Proximal phalanx fractures are common injuries of the hand, with estimated rates of 68 injuries per 100,000 persons per year. The highest incidence was reported in males between the ages of 30 and 57 years. The fifth finger is most frequently affected at 38% [2]. Fractures with fragment displacement should be repaired as soon as possible post-injury; however, this does not guarantee the restoration of the finger's pre-injury function [3].

One of the main factors contributing to the development of osteoarthritis is trauma. Post-traumatic osteoarthritis accounts for approximately 12% of all osteoarthritis cases [4]. Sometimes, patients may not even associate their disease with a previous injury. A thorough interview can reveal a history of joint trauma in some patients with initially non-traumatic osteoarthritis. A key element of post-traumatic osteoarthritis treatment is not only pain relief but also the return of joint function close to pre-injury levels. This is particularly important for young and physically active individuals. Unfortunately, the ability to return to satisfactory PIP joint mobility after surgery is often limited.

There are several effective surgical methods for treating this condition, including arthrotomy with a Reg-Joint implant, arthroplasty using vascularized toe grafts, arthroplasty using non-vascularized grafts, and endoprosthetics using various implants, such as silicone prostheses, resurfaced metal prostheses, and hemi- or total prostheses. These methods are quite effective in relieving pain. Some of them may have limitations due to the difficulty and experience of the surgeon, as well as higher costs. An additional challenge in the treatment of post-traumatic arthritis is the often very limited ability to return the PIP joint to a satisfactory range of motion [5-8]. Current evidence-based medicine reports indicate that no ideal treatment method exists that combines complete pain relief with the preservation of the preoperative range of motion [3].

## Case presentation

**Figure 1:**
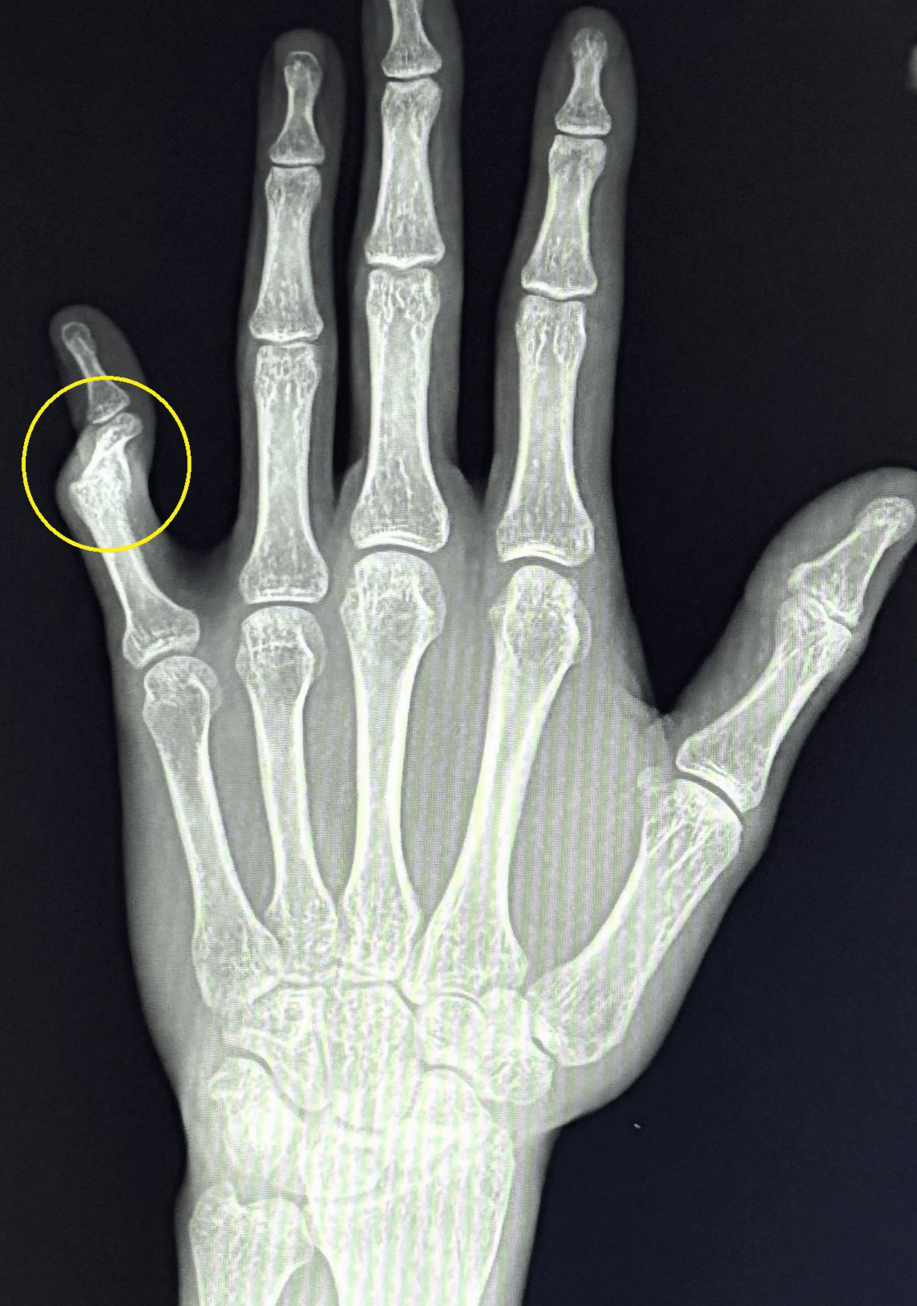
Boutonniere PIP deformity of finger 5 in A-P projection PIP: proximal interphalangeal joint

**Figure 2:**
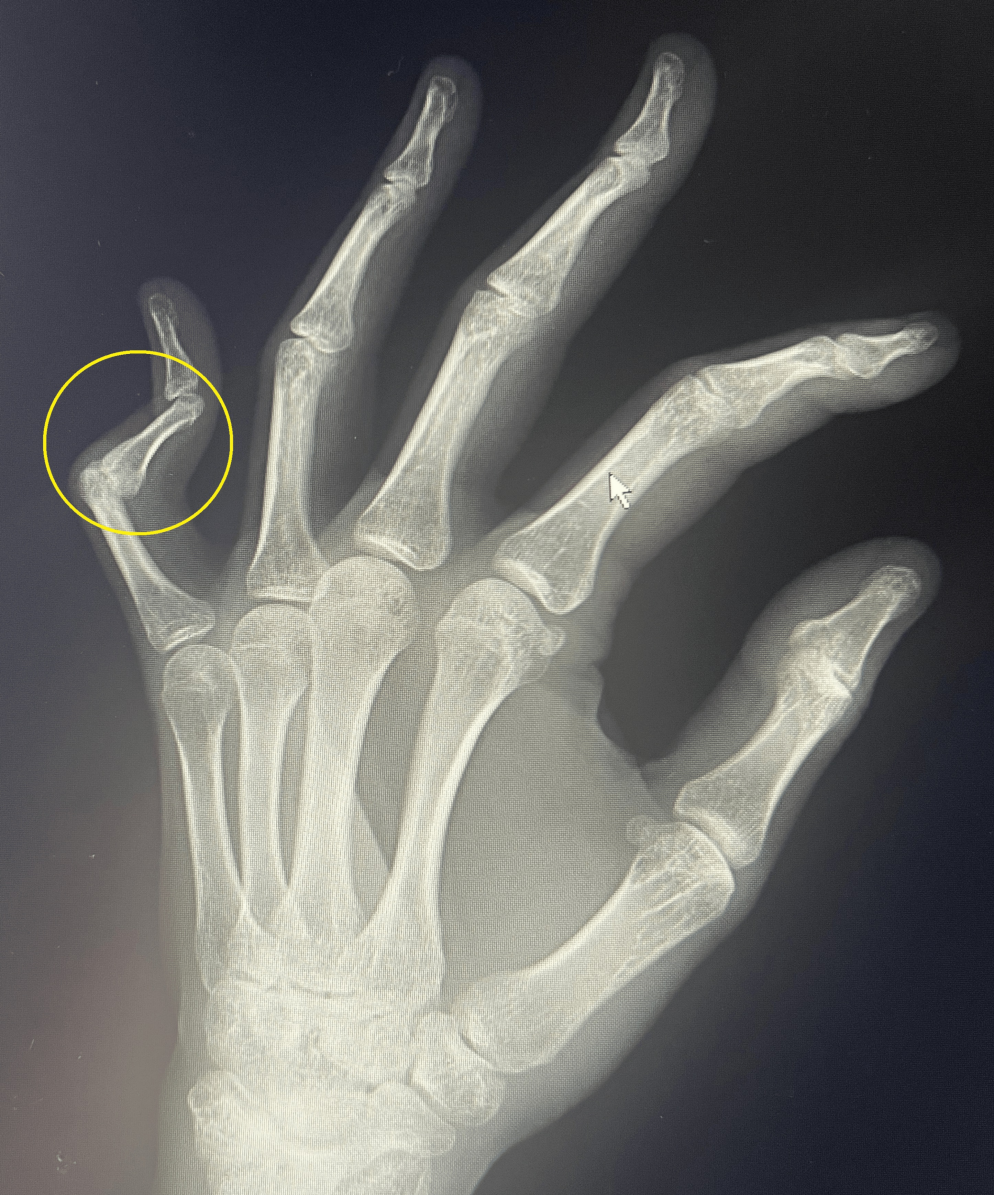
Boutonniere PIP deformity of finger 5 in Robert's projection PIP: proximal interphalangeal joint

This article describes the treatment of a young, active female patient who, several years ago, suffered a PIP joint injury in her left fifth finger following a fall from a height of 2 m. Initially, the patient was treated at another hospital with open reduction internal fixation (ORIF) and stabilization with two Kirschner wires. Twelve months after the injury, the patient was referred to our clinic. A complication of this treatment was a "boutonniere" finger deformity with stiffening of the phalanx in the position of flexion contracture of 80° and the presence of finger pain with a numerical rating scale (NRS) score of 6 [9]. The functional efficiency of the limb assessed using the Disabilities of the Arm, Shoulder and Hand Questionnaire (DASH) questionnaire was 72.2 [10]. The power grip strength assessed using a Saehan Hydraulic Hand Dynamometer (Fabrication Enterprises, White Plains, NY, USA) was 9 kg. An X-ray taken during the first visit confirmed initial degenerative changes in the joint surface of the proximal phalanx of the left fifth finger (Figures 1, 2).

**Figure 3:**
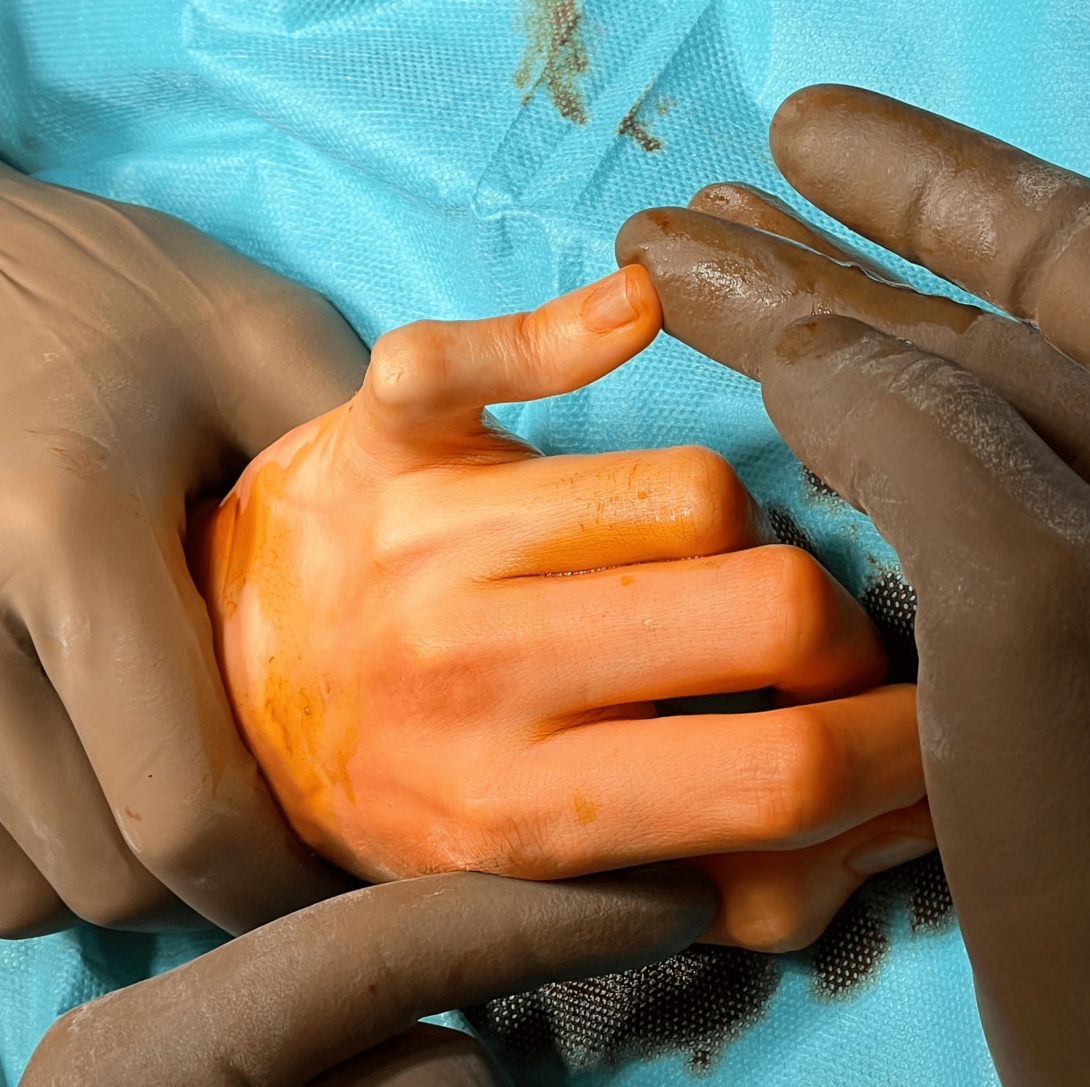
Contracture and boutonniere deformity of the V finger

Based on the clinical examination and X-ray, the patient was qualified for individually planned surgical treatment, joint replacement using a cementless CapFlex hemiarthroplasty. The procedure was performed via a volar approach, using an oblique incision at the level of the proximal and middle phalanges of the fifth finger. After careful dissection of the flexors, the flexor digitorum profundus, flexor digitorum superficialis, and the volar plate of the fifth finger of the left hand, the PIP joint was approached and dislocated using the shotgun technique. Degenerative changes in the distal joint surface of the proximal phalanx and moderate degenerative changes in the joint surface of the middle phalanx of the fifth finger were confirmed. After preparing the proximal joint surface, the proximal component of the CapoFlex prosthesis was implanted. The PIP joint was repositioned, ensuring stability. Intraoperative X-ray confirmed proper preparation of the joint surfaces and the position of the prosthetic implant. The PIP joint capsule was sutured, and a volar plate repair of the fifth finger was performed. Layered sutures and a sterile dressing were applied. A volar plaster cast was implanted for 10 days, encompassing the fourth and fifth fingers of the left hand. After 10 days, the skin sutures and plaster splint were removed, and rehabilitation began, aiming to restore the normal range of motion and functional capacity of the operated hand. The patient was placed on a home rehabilitation program. Initially, it included passive and active exercises for both the operated finger and the other fingers, with particular emphasis on flexor tendon gliding exercises. Over time, resistance exercises and activities of daily living were introduced. All recommended exercises were performed after prior physiotherapy instruction, which was performed during a follow-up visit to the orthopaedic clinic. A follow-up examination was performed after six months, which revealed an unsatisfactory clinical outcome because the total range of motion was 10° (extension deficit: 80°; flexion range: 90°), and the boutonniere deformity was still present (Figure 3). Pain intensity was 0 on the NRS scale. The functional efficiency of the limb in DASH improved slightly, obtaining a value of 64.4, as did the global grip strength, which obtained 11 kg.

**Figure 4:**
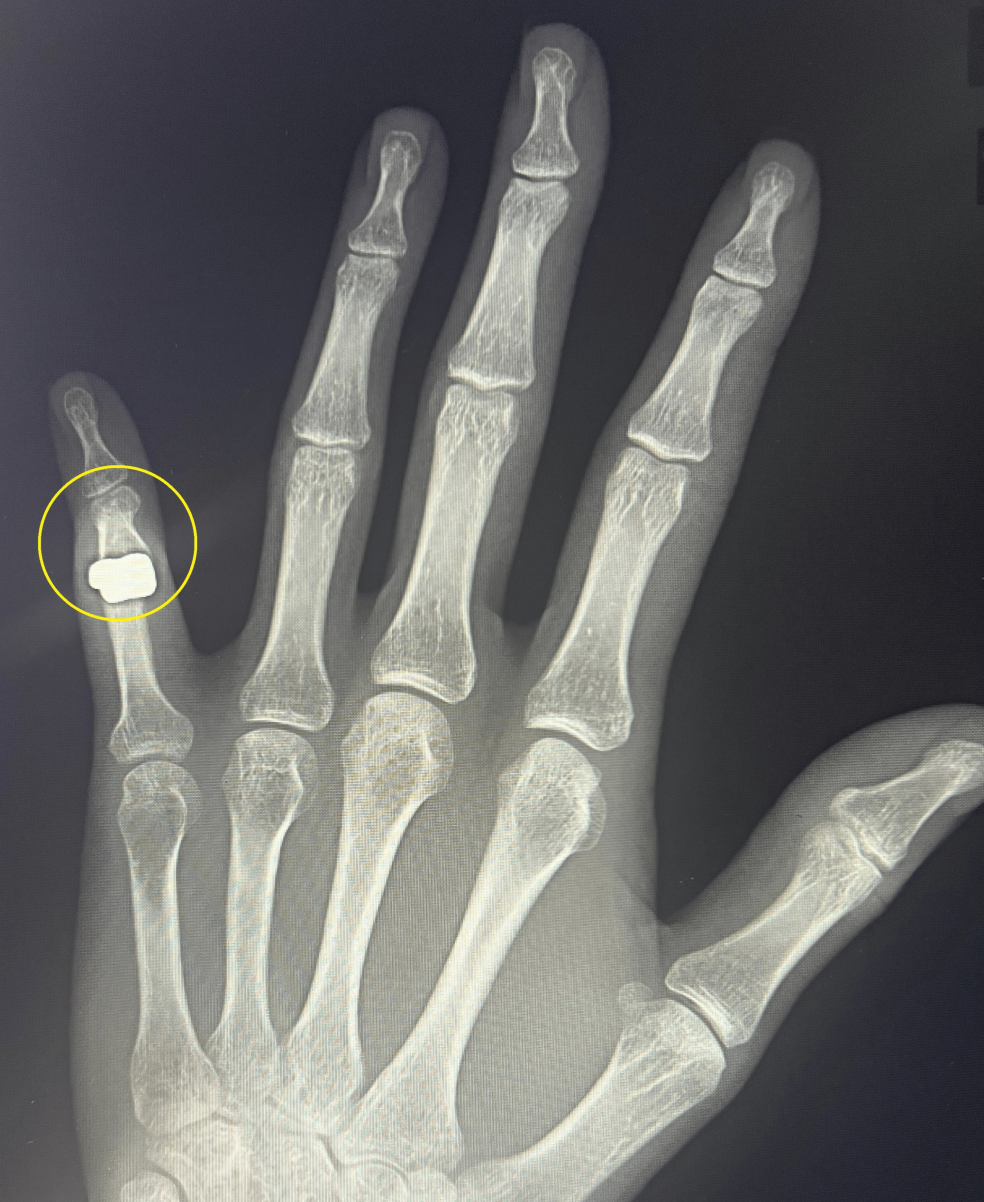
Control X-ray six months after implantation of the CapFlex cementless hemiprosthesis in the A-P projection A-P: anteroposterior

**Figure 5:**
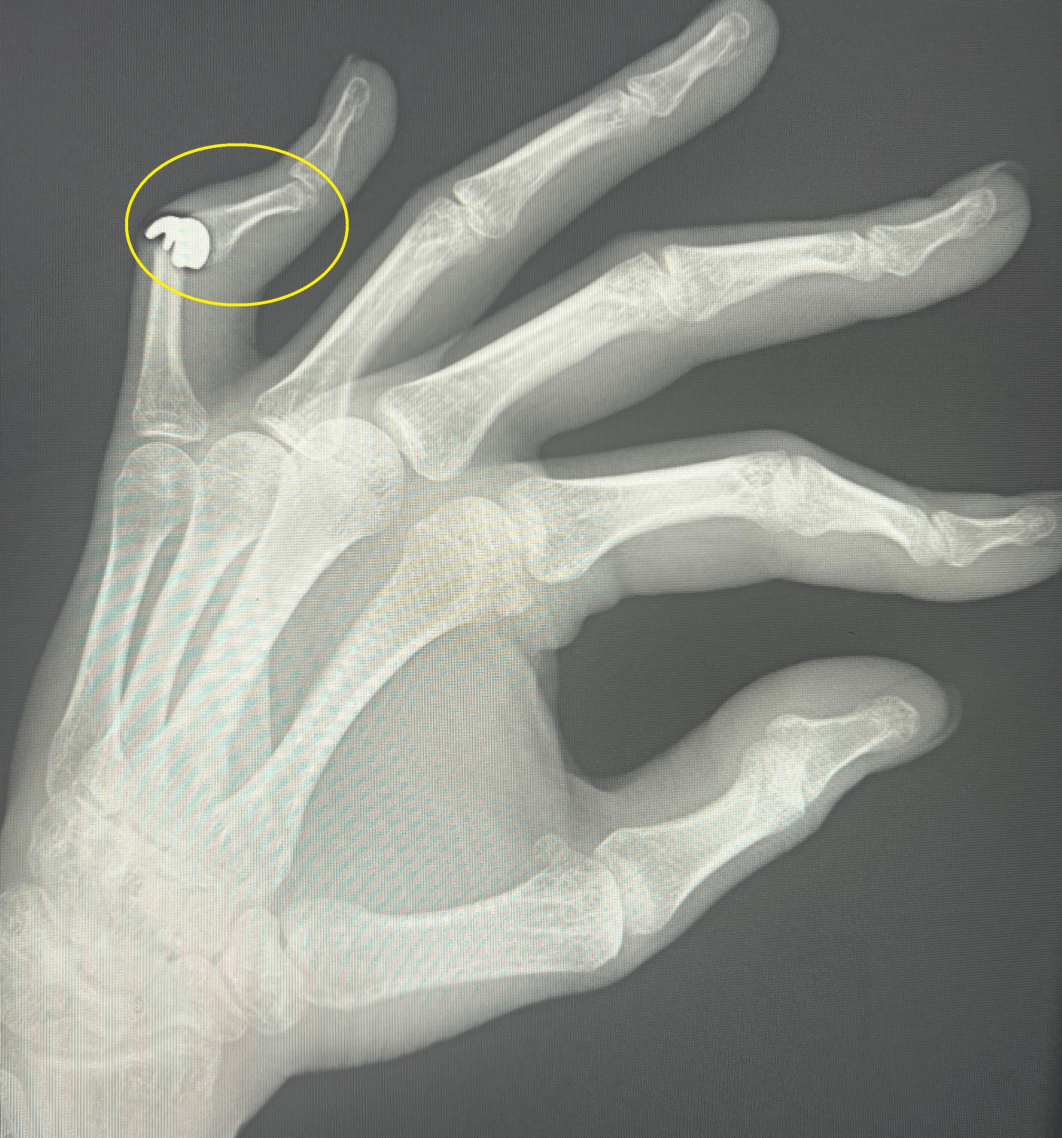
Control X-ray six months after implantation of the CapFlex cementless hemiprosthesis in Robert's projection

A follow-up X-ray revealed slight improvement in the boutonniere deformity with no evidence of implant loosening (Figures 4, 5). Due to the patient's active lifestyle, a joint decision was made to qualify the patient for revision surgery using a 3S ORTHO semi-constrained prosthesis.

**Figure 6:**
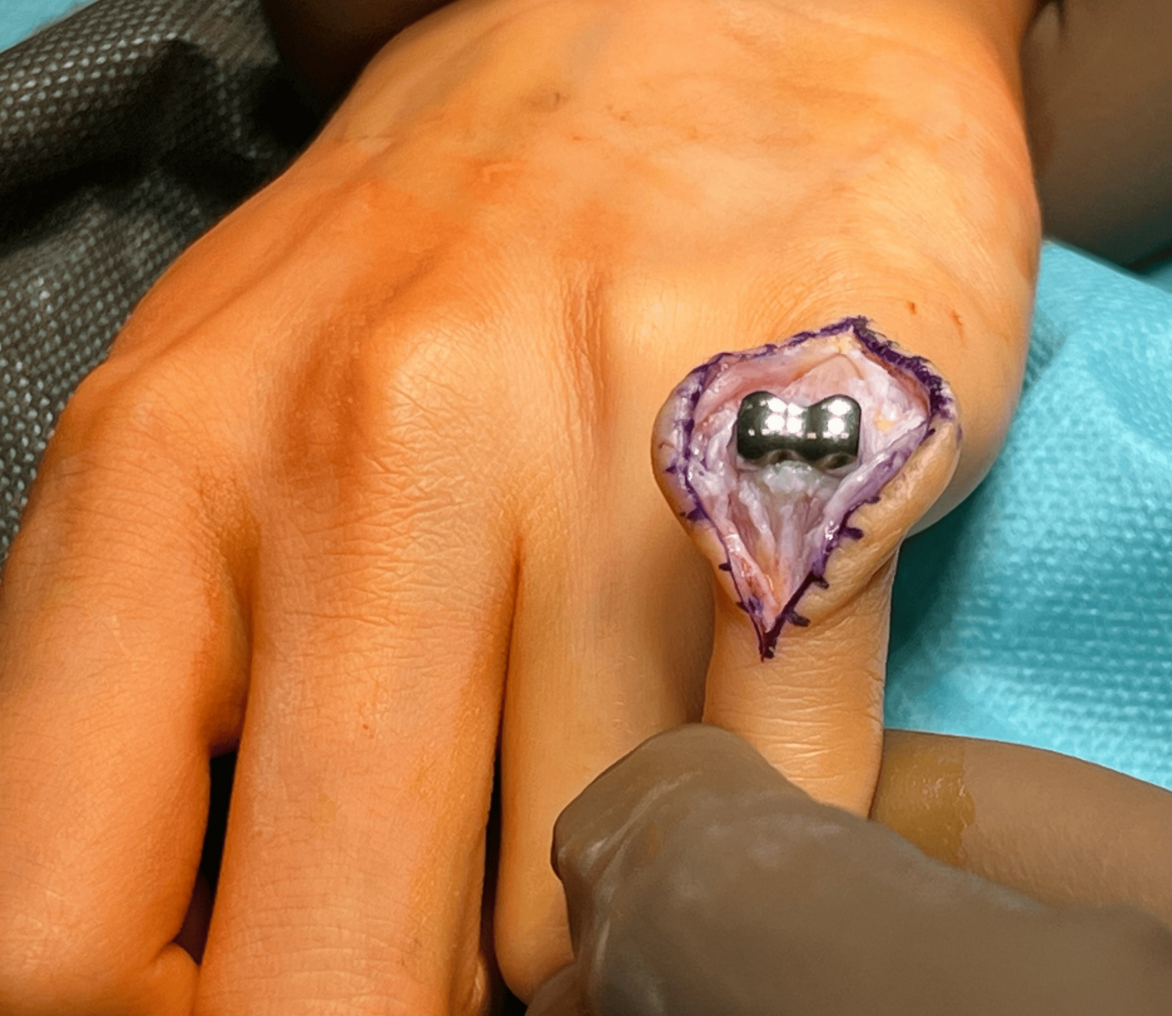
Tissue dissection above the PIP joint with visualization of the CapFlex prosthesis PIP: proximal interphalangeal joint

Revision surgery, consisting of the removal of the CapFlex prosthesis and the implantation of the 3S ORTHO prosthesis, was performed six months after the follow-up visit. The procedure was performed via a dorsal approach using a dorsal oblique incision at the level of the proximal and middle phalanges of the fifth finger (Figure 6).

**Figure 7:**
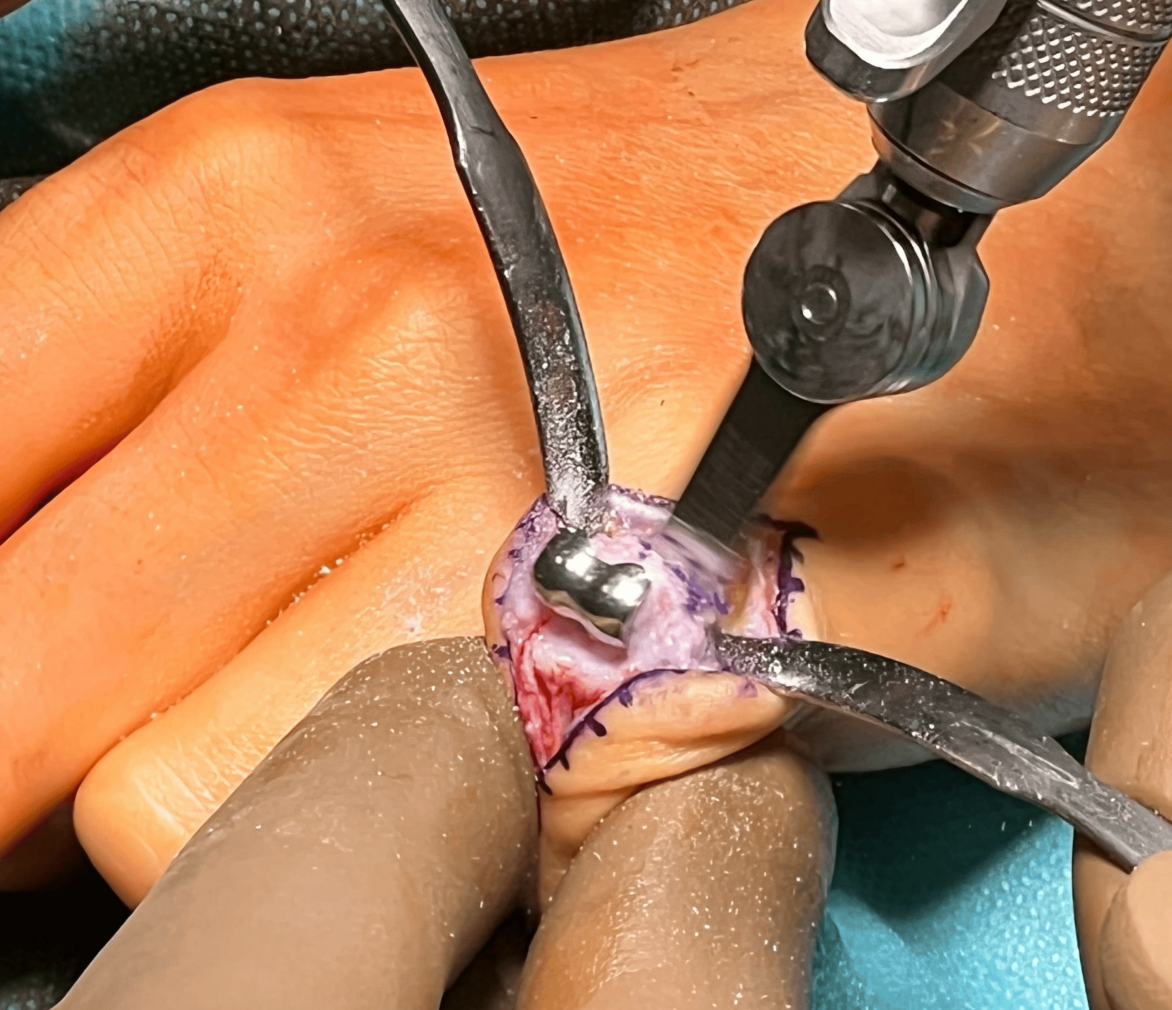
Removal of the CapFlex prosthesis

**Figure 8:**
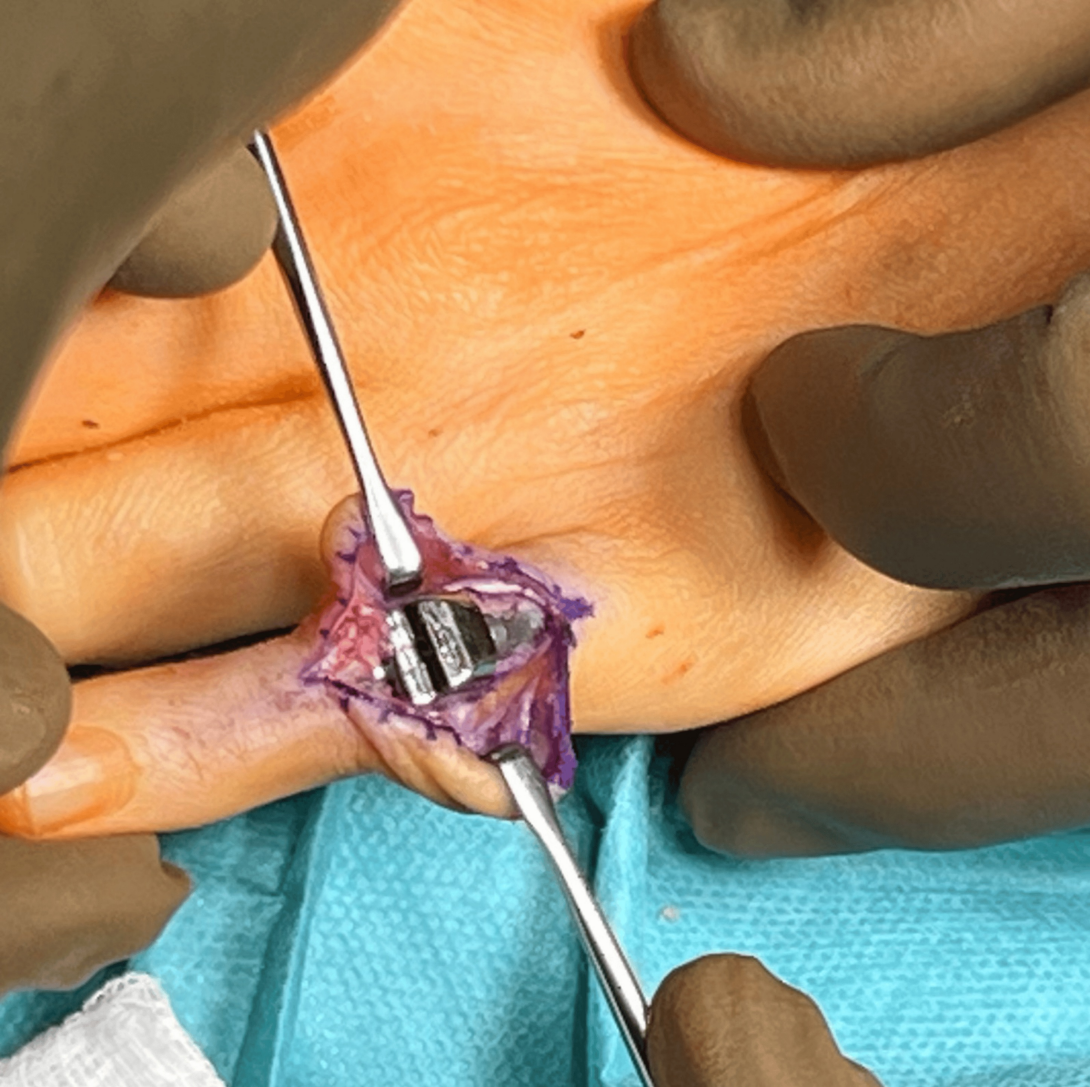
A properly implanted 3S ORTHO prosthesis

After thorough tissue dissection, the central band was reached and cut longitudinally to reach the joint capsule of the PIP joint. The PIP joint capsule was also cut longitudinally, revealing a properly implanted proximal component of the CapFlex prosthesis, implanted in the proximal phalanx of the finger, and the degeneratively altered joint surface of the middle phalanx. Using a special trimmer, the proximal phalanx and the CapFlex prosthesis component were trimmed, and the joint surfaces of the proximal and middle phalanges were then prepared (Figures 7, 8).

**Figure 9:**
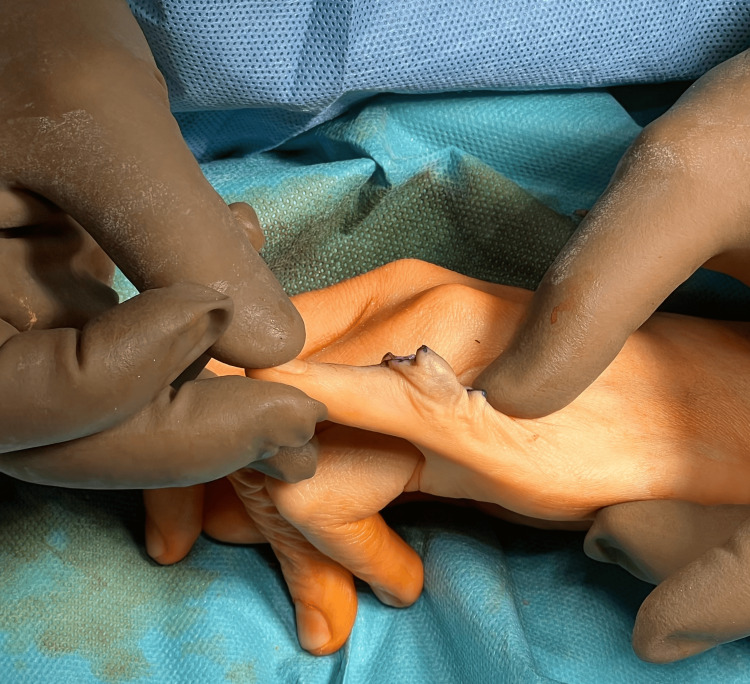
Intraoperative assessment of joint stability

In the next stage, screws were implanted to secure the prosthesis to the proximal and middle phalanges and a 3D ORTHO semi-constrained prosthesis connector was introduced, obtaining a stable PIP joint (Figure 9).

**Figure 10:**
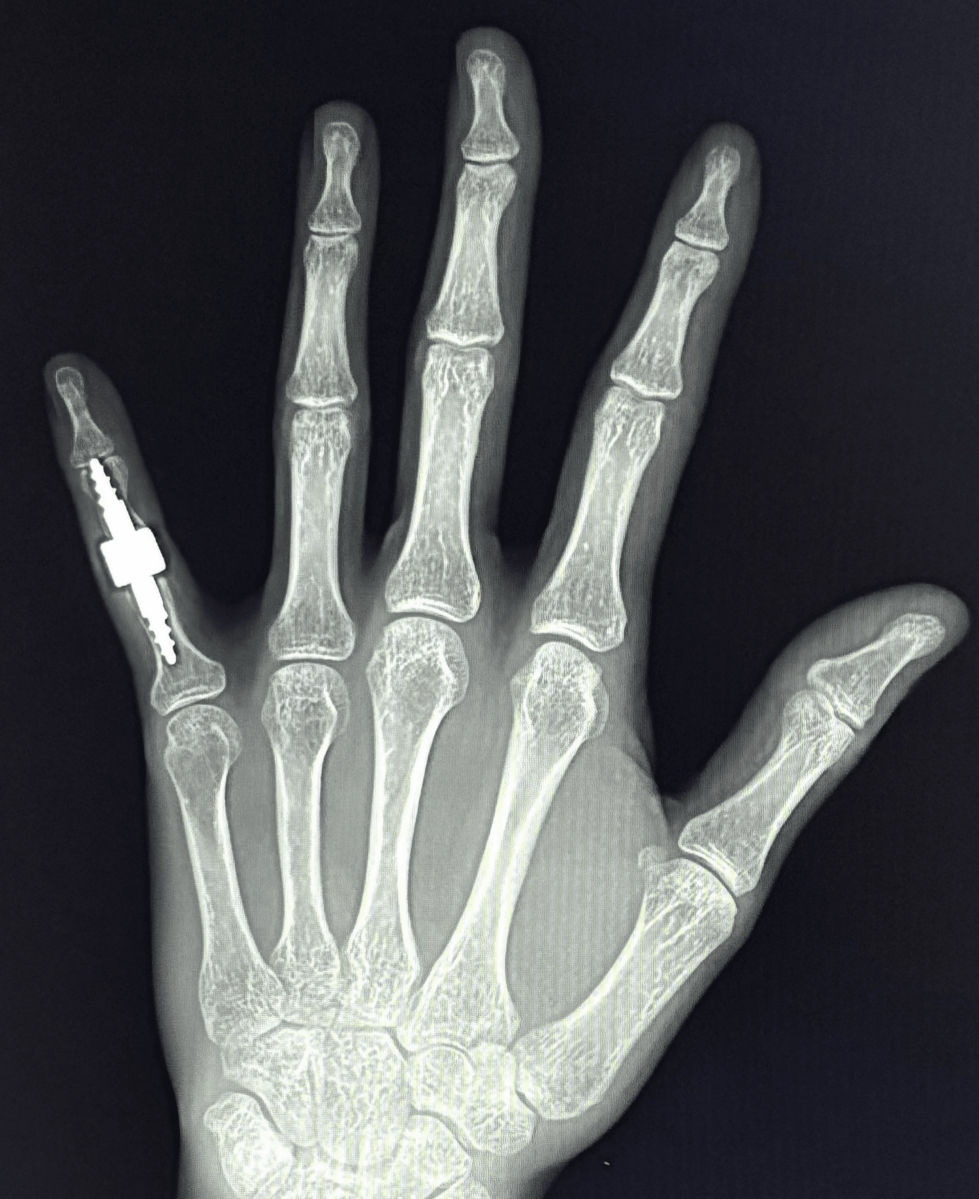
Control X-ray eight weeks after implantation of the 3S ORTHO prosthesis in the A-P projection A-P: anteroposterior

**Figure 11:**
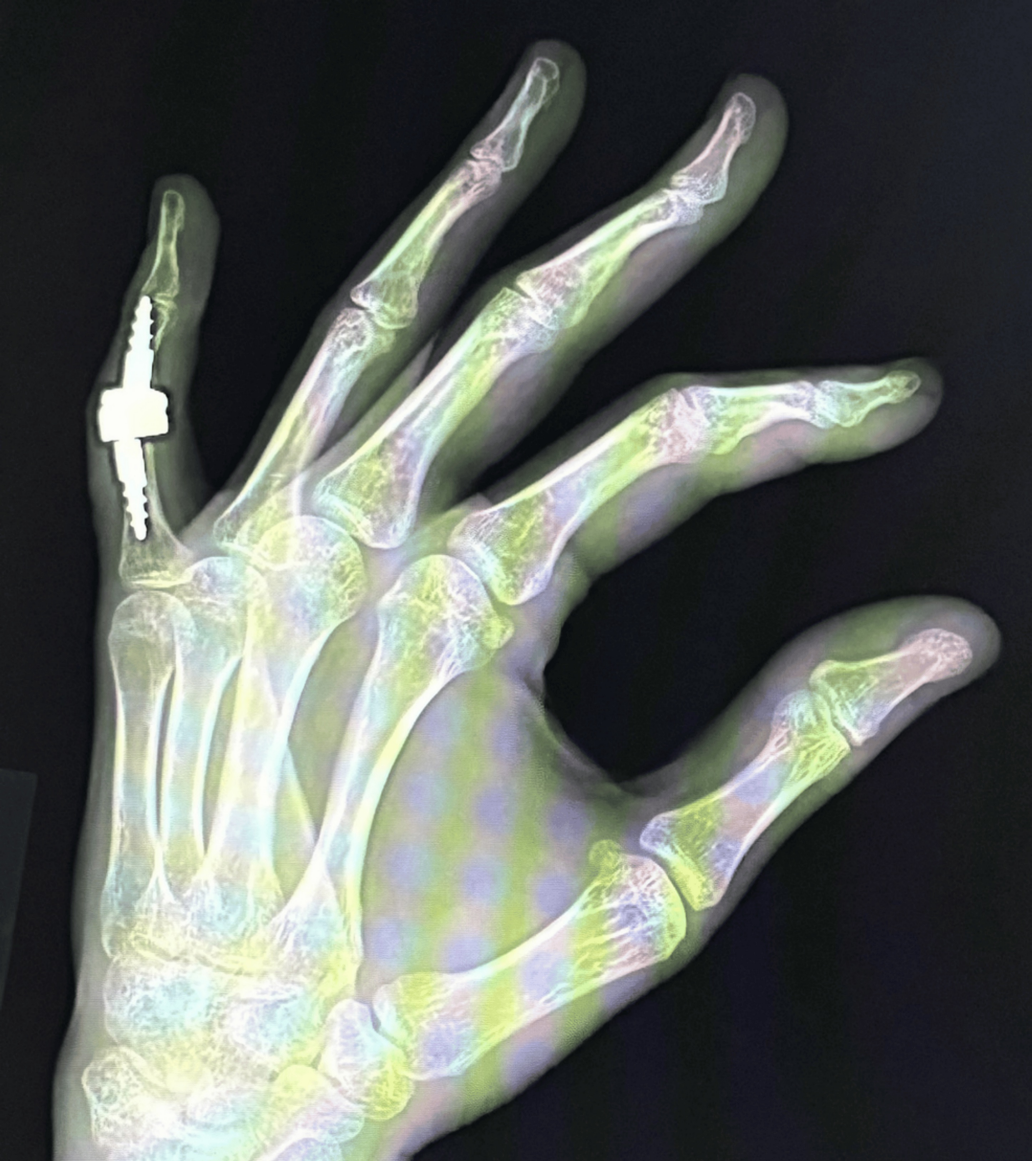
Control X-ray eight weeks after implantation of the 3S ORTHO prosthesis in Robert's projection

Intraoperative X-ray confirmed proper preparation of the phalangeal joint surfaces and the correct positioning of the prosthetic implants. The PIP joint capsule was sutured. Layered sutures and a sterile dressing were applied. A palmar plaster splint was implanted for 10 days, encompassing the fourth and fifth fingers of the left hand. On day 10, the sutures and plaster splint were removed, and rehabilitation began to restore the normal range of motion. The rehabilitation regimen was the same as for the previous implantation of the uncemented CapFlex hemiprosthesis. A follow-up examination performed eight weeks after the surgery showed correction of the butenoid deformity and improvement of the flexion range of motion to 50°s, with limitation of the active extension movement (extension deficit reached 15°). Pain intensity was rated 0 on the NRS scale. Additionally, a significant improvement in the functional efficiency of the limb (DASH: 29.8), and the global grip strength value to the level of 17 kg was observed. A follow-up X-ray examination revealed the prosthesis was in proper position, with no signs of loosening of any of its components (Figures 10, 11).

**Figure 12:**
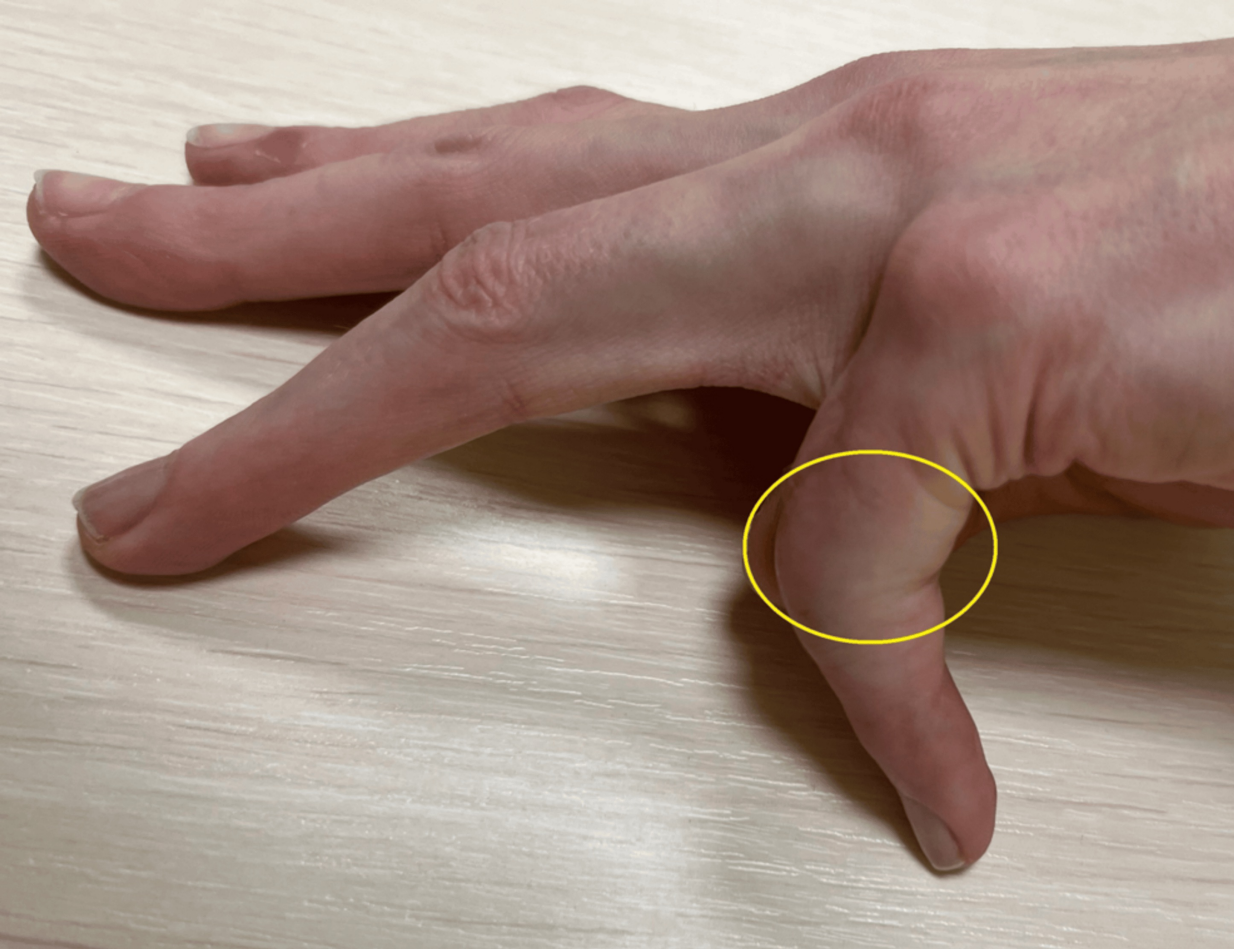
Active flexion range of the PIP joint PIP: proximal interphalangeal joint

**Figure 13:**
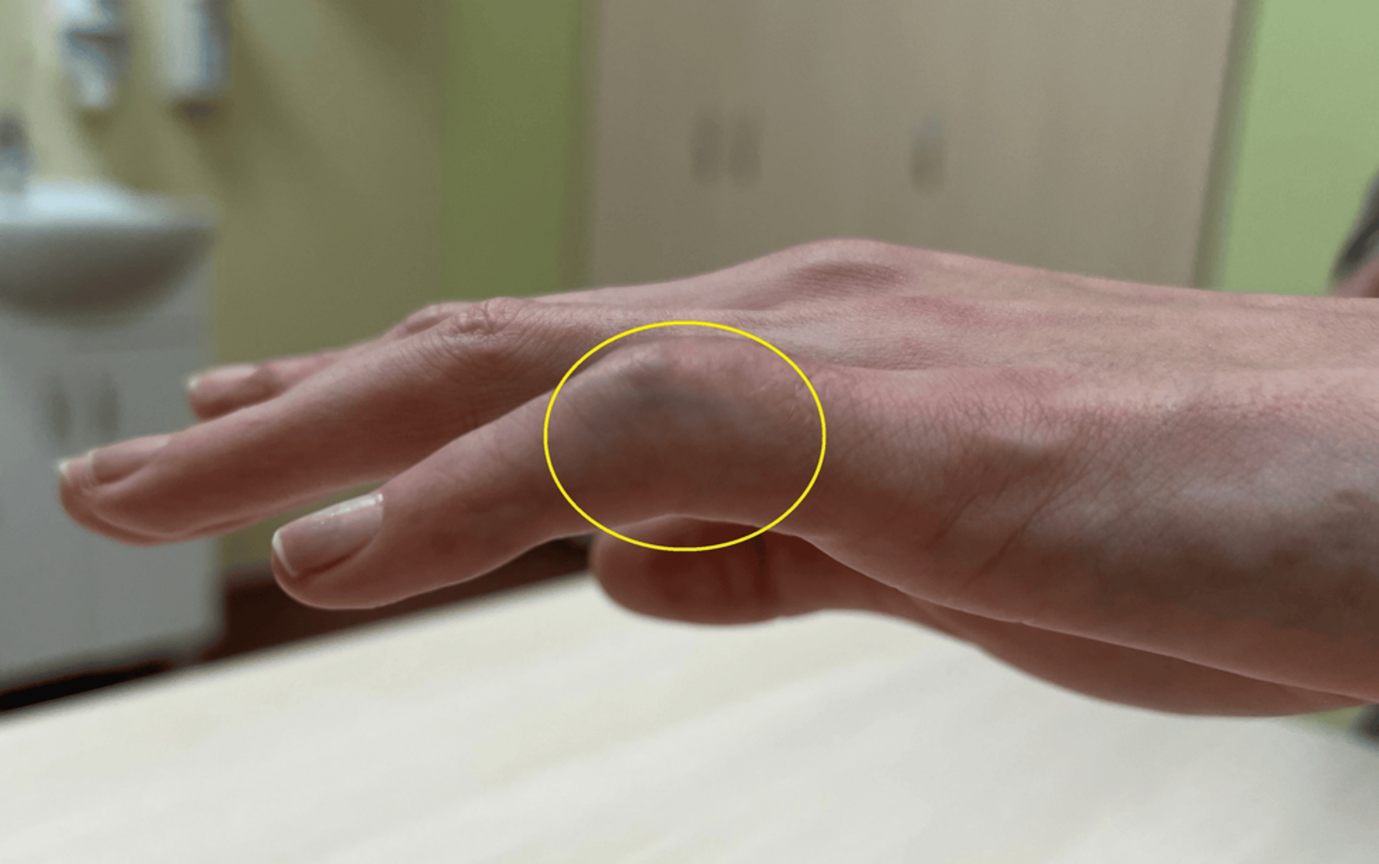
Active extension range of the PIP joint PIP: proximal interphalangeal joint

After the follow-up examination, the patient was referred for additional specialized physiotherapy treatments, including post-isometric muscle relaxation, manipulation exercises, and whirlpool. Additional physiotherapy treatments resulted in further improvement in the range of motion and functional efficiency of the DASH 23.4 limb. The obtained active range of motion of the operated finger is shown in Figures 12, 13. However, no further improvement in grip strength was observed.

The recovered limb function met the patient's expectations and allowed her to return to previously performed activities, such as playing tennis and riding a motorcycle. Detailed results obtained during the individual follow-up periods are presented in Table 1.

**Table 1:** Results of the assessed parameters in subsequent observation periods NRS: numerical rating scale; DASH: Disabilities of the Arm, Shoulder, and Hand; PIP: proximal interphalangeal point NRS, assessing the intensity of resting pain; range of motion in the sagittal plane, assessed using a goniometer; global grip strength, assessed using the SAEHAN hydraulic dynamometer (SAEHAN Corporation, Gyeongsangnam-do, Republic of Korea); DASH, assessing the functional status of the upper limb

Treatment stage	NRS	Range of motion (extension/flexion)	Total range of motion	DASH	Global grip strength	Boutonniere PIP deformity
12 months after ORIF with Kirschner wires	6	Stiffening of the joint in the flexion position of 80°	0°	72.2 (100)	9	Yes
6 months after CapFlex hemiarthroplasty	0	Extension deficit: 80° flexion range: 90°	10°	63.4 (100)	11	Partial improvement
8 weeks 3S ORTHO semi-constrained prosthesis	0	Extension deficit: 15° flexion range: 50°	35°	29.8 (100)	17	no
After additional specialized physiotherapy treatments	0	Extension deficit: 15° flexion range: 60°	45°	23.4 (100)	17	no

## Discussion

In the Introduction, we noted that there are several surgical methods for treating post-traumatic osteoarthritis of the PIP joint. Each of these methods is effective in reducing pain. However, a problem clinicians encounter daily is that some of them limit the ability to return the PIP joint to a satisfactory range of motion [5-8].

Limited range of motion in a joint limits the ability to perform daily activities, not to mention increased professional or recreational activity. Unfortunately, not every patient is eligible for techniques that help restore the optimal range of motion in the joint. The choice of treatment method depends on the patient's age, the severity of the degenerative disease, comorbidities, economic factors, and the patient's expectations regarding their activity. In the case of the latter factor, endoprosthetic replacement offers the greatest chance of restoring the normal range of motion. However, it should be remembered that, financially, this is the most expensive procedure and does not always provide the expected result (e.g., our patient who, after implantation of a cementless CapFlex hemiarthroplasty, had to undergo a revision procedure using 3S ORTHO, after which we observed an early, satisfactory result). In fact, after implantation of an endoprosthesis, the risk of complications should always be taken into account. It is also worth mentioning that each implanted endoprosthesis has a survival time, which carries the risk of a revision procedure in the future. Wagner et al. report that PIP joint arthroplasty procedures achieve approximately 75% survival after 14 years, but 25% require a revision procedure. The need for secondary revision surgery of PIP joints using various implants occurs in approximately 30% of cases within five years [11]. The material and type of prosthesis used for arthroplasty certainly influence implant durability. Silicone implants have poor postoperative stability and are more likely to fail. Resurface prostheses made of various materials, such as pyrocarbon, titanium, or ceramic, have a higher rate of postoperative complications [12,13].

Describing the main causes of revision surgery, it is worth mentioning that complications can be divided into early postoperative and long-term, including infection, aseptic loosening, implant failure, periprosthetic fractures, dislocations, or joint contractures. Some of these complications absolutely require surgical repair. These most commonly include infection, fracture, or implant damage [14,15]. PIP joint contracture in a patient after joint replacement is not an absolute indication for revision surgery, as pain relief remains the most important treatment outcome. However, in young patients, especially those actively participating in sports, the return to mobility plays a crucial role. Implantation of a PIP joint replacement certainly requires a higher financial outlay and is not always successful, but it offers a chance. The choice of treatment method in such a situation also depends on the surgeon's preferences, experience, and capabilities.

## Conclusions

Arthroplasty of the PIP joint using a cementless CapFlex hemiarthroplasty corrected the boutonniere deformity. At follow-up, a significant degree of flexion contracture developed. Revision surgery involving implantation of a 3S ORTHO prosthesis improved the flexion range of motion. The extension range of motion did not return to normal. According to the patient, the regained hand function met her expectations and allowed her to return to her previously performed activities. These results reflect the early follow-up period.

## References

[1] Binstead JT, Tafti D, Hatcher JD (2025). Boutonniere Deformity. https://www.ncbi.nlm.nih.gov/books/NBK470323/.

[2] Petrovych O, Florek J, Georgiew F, Kawa P, Florek P (2025). Comminuted transarticular fracture of the middle phalanx: a non-standard surgical procedure. Cureus.

[3] Florek J, Georgiew F, Petrovych O (2024). The hemiarthroplasty of the proximal interphalangeal joint in post-traumatic degenerative disease: a case report. Cureus.

[4] Punzi L, Galozzi P, Luisetto R, Favero M, Ramonda R, Oliviero F, Scanu A (2016). Post-traumatic arthritis: overview on pathogenic mechanisms and role of inflammation. RMD Open.

[5] Florek J, Georgiew F, Szklany K, Kotela I (2020). Evaluating outcomes of surgical treatment of patients with degenerative changes in the carpometacarpal joint of the thumb using percutaneous stabilization with Herbert screws and Reg-Joint implants-a pilot study. Chir Narzadow Ruchu Ortop Pol.

[6] Dautel G (2018). Vascularized toe joint transfers to the hand for PIP or MCP reconstruction. Hand Surg Rehabil.

[7] Frueh FS, Calcagni M, Lindenblatt N (2015). The hemi-hamate autograft arthroplasty in proximal interphalangeal joint reconstruction: a systematic review. J Hand Surg Eur Vol.

[8] Darwish I, Imani S, Baba M (2023). Prosthesis options for proximal interphalangeal joint arthroplasty in osteoarthritis: a systematic review and meta-analysis. J Hand Surg Asian Pac Vol.

[9] Nugent SM, Lovejoy TI, Shull S, Dobscha SK, Morasco BJ (2021). Associations of pain numeric rating scale scores collected during usual care with research administered patient reported pain outcomes. Pain Med.

[10] Golicki D, Krzysiak M, Strzelczyk P (2014). Translation and cultural adaptation of the Polish version of the Disabilities of the Arm, Shoulder and Hand (DASH) and QuickDASH questionnaires. Ortop Traumatol Rehabil.

[11] Wagner ER, Luo TD, Houdek MT, Kor DJ, Moran SL, Rizzo M (2015). Revision proximal interphalangeal arthroplasty: an outcome analysis of 75 consecutive cases. J Hand Surg Am.

[12] Herren D (2019). The proximal interphalangeal joint: arthritis and deformity. EFORT Open Rev.

[13] Reischenböck V, Marks M, Herren DB, Schindele S (2021). Surface replacing arthroplasty of the proximal interphalangeal joint using the CapFlex-PIP implant: a prospective study with 5-year outcomes. J Hand Surg Eur Vol.

[14] Helder O, Marks M, Schweizer A, Herren DB, Schindele S (2021). Complications after surface replacing and silicone PIP arthroplasty: an analysis of 703 implants. Arch Orthop Trauma Surg.

[15] Forster N, Schindele S, Audigé L, Marks M (2018). Complications, reoperations and revisions after proximal interphalangeal joint arthroplasty: a systematic review and meta-analysis. J Hand Surg Eur Vol.

